# Influenza D Virus in Black Donkeys, Northern China

**DOI:** 10.3201/eid3112.250666

**Published:** 2025-12

**Authors:** Mingshuai Shen, Jieshi Yu, Bin Fu, Cuilian Yu, Chunqi Cui, Shumin Chen, Cun Liu, Kezhou Wang, Zhao Wang

**Affiliations:** Shandong First Medical University, Jinan, China (M. Shen, B. Fu, C. Yu, C. Cui, K. Wang, Z. Wang); Agro-biological Gene Research Center of Guangdong Academy of Agricultural Sciences, Guangzhou, China (J. Yu); Shandong Provincial Center for Animal Disease Control, Jinan (S. Chen, C. Liu)

**Keywords:** influenza, viruses, respiratory infections, zoonoses, influenza D virus, black donkeys, serologic tests, China

## Abstract

Influenza D virus (IDV) is prevalent in cattle in China, and a risk for spillover to other species exists. We detected IDV antibodies in 6/315 of black donkeys in northern China, suggesting cattle-to-donkey transmission and demonstrating the expanding host range of IDV and the need for reassessment of cross-species transmission risks.

Since its initial isolation from a pig with respiratory symptoms in 2011, influenza D virus (IDV) has been documented in cattle, small ruminants (e.g., sheep), dogs, camels, and wild ungulates (*1*–*3*). Similar to other influenza viruses, IDV carries genetic traits that might aid in host range expansion and virulence enhancement; genetic adaptations frequently occur during cross-species transmission events (*4*). 

Donkeys are economically important livestock species in China. Because of the widespread distribution and high seroprevalence of IDV in cattle in China (*5*), along with the frequent interactions among domestic animals in rural areas of the country, investigating donkey exposure to IDV is essential to assess the virus’s risk to animal health.

This study used a cross-sectional design. A total of 315 serum samples were randomly collected from black donkeys (*Equus asinus*, a local breed) across 9 commercial farms in northern China. Sampling was conducted regardless of age or sex. All serum specimens were preserved at –80°C and tested within 6 months of collection. C.L. provided all samples. We obtained receptor-destroying enzyme (RDE) from Denka Seiken Co. Ltd (https://denka-seiken.com), and we purchased chicken red blood cells from Wuhan Keyi Biotechnology Co. Ltd (https://m.labbase.net/CompanyIndex-16219.html). J.Y. provided MDCK cells, which we maintained in Dulbecco’s modified Eagle medium supplemented with 10% fetal bovine serum. J.Y. provided all viral strains used in this study, which included IDV (D/bovine/CHN/JY3002/2022), influenza A virus (A/People’s Republic of China/SWL1304/2023), and influenza B virus (B/Guangdong/266/2021). For hemagglutination inhibition (HI) assays, we treated serum samples with RDE (1:3 ratio, 37°C for 18–20 hours, 56°C for 30–60 minutes) (*6*).

All animal procedures in this study were reviewed and approved by the Animal Ethics Committee of Shandong First Medical University (approval no. W202502250029). Sampling was conducted under veterinary supervision in accordance with national guidelines for the care and use of animals.

We mixed viral antigen (4HAU) with serially diluted serum samples (1:10 starting in phosphate-buffered saline in V-bottom plates), incubated them at 37°C for 30 minutes, and assessed them with 0.5% chicken red blood cells at room temperature for 15 minutes. HI titers were the highest dilution inhibiting hemagglutination (we defined a titer >10 as positive). The HI >10 cutoff was validated in prior IDV studies to balance sensitivity and specificity; using a higher cutoff generally lowers apparent seroprevalence, whereas a lower cutoff increases sensitivity and can raise estimated prevalence (*7*).

Virus neutralization (VN) assays used IDV (100 tissue culture infectious dose 50). We mixed RDE-treated serum specimens (2-fold dilutions in Dulbecco’s modified Eagle medium from 1:10) with virus at 37°C for 1 hour, inoculated them onto MDCK cells, and observed them for cytopathic effect at 37° C in 5% CO_2_ for 48–72 hours. Neutralizing titers were the reciprocal of the highest dilution showing complete cytopathic effect inhibition (we defined a titer >10 as positive) (*8*).

Among 315 donkey serum samples collected from 9 distinct farms in northern China, 6 (1.9%, 95% CI 0.7%–4.1%; p = 0.006) were HI-positive for IDV; 3 of those 6 were positive samples exhibiting HI titers of 40. Furthermore, 5 HI-positive samples were confirmed by virus neutralization (VN) assays, demonstrating VN titers ranging from 10 to 40. We detected no influenza A virus or influenza B virus antibodies (Table). Geographically, positive samples were clustered in farms in 2 different cities: 1 farm showed a seroprevalence of 3.07% (2 of 65 samples), and the other exhibited a higher rate of 4.08% (4 of 98 samples).

**Table:** HI and VN antibody titers against IDV in serum samples from black donkeys, northern China, 2025*

Sample no.	IAV (A/People’s Republic of China/SWL1304/2023[H1N1]), HI	IBV (B/Guangdong/266/2021), HI	IDV (D/bovine/CHN/JY3002/2022), IDV	
			HI	VN
1646	<10	<10	10, 10	10, 10
1714	<10	<10	10, 10	<10
1116	<10	<10	20, 20	20, 20
842	<10	<10	40, 40	20, 20
558	<10	<10	40, 40	20, 40
35	<10	<10	40, 40	20, 20

*Samples were tested against 3 strains: IAV, IBV, and IDV. Titers below the detectable threshold (10) were indicated as <10 and considered negative. HI, hemagglutination inhibition test; IAV, influenza A virus; IBV, influenza B virus; IDV, influenza D virus; VN, virus neutralization.

Although the 1.9% infection rate was substantially lower than that reported in cattle (77.5%) (*9*), our findings indicate that donkeys might be a novel host species. This development expands the known host range of IDV and underscores the need to reassess its cross-species transmission potential. This study is limited by its small sample size, restricted geographic coverage, and the lack of detailed farm management data, which prevented assessment of potential risk factors. To address those gaps, future work should include broader serologic surveys, genomic surveillance, and targeted studies in mixed farming systems. A previous study reported IDV antibody prevalence in horses in the United States (*10*). Together with our finding in donkeys, that finding suggests that multiple equid species might serve as IDV hosts, broadening the ecologic and epidemiologic understanding of IDV. These results highlight the need for continued One Health surveillance and reassessment of cross-species transmission risks.
